# Ambient Odor Exposure Affects Food Intake and Sensory Specific Appetite in Obese Women

**DOI:** 10.3389/fpsyg.2019.00007

**Published:** 2019-01-15

**Authors:** Cristina Proserpio, Cecilia Invitti, Sanne Boesveldt, Lucia Pasqualinotto, Monica Laureati, Camilla Cattaneo, Ella Pagliarini

**Affiliations:** ^1^Department of Food, Environmental and Nutritional Sciences, University of Milan, Milan, Italy; ^2^Department of Medical Sciences and Rehabilitation, IRCCS Istituto Auxologico Italiano, Milan, Italy; ^3^Division of Human Nutrition and Health, Wageningen University & Research, Wageningen, Netherlands

**Keywords:** appetite, eating behavior, food consumption, food cues, overweight, smell

## Abstract

Food odors are important in food perception not only during consumption, but also in anticipation of food. Even though it is well established that smell is involved in eating behavior, its role in affecting actual food consumption is still unclear, especially in morbidly obese subjects, who are reported to be more affected by sensory cues than lean subjects. The aim of the present study was to investigate the influence of ambient odor exposure on *ad libitum* food intake and on sensory specific appetite in obese women. Thirty obese women (BMI: 34.9 ± 0.8 kg m^-2^; age: 50.8 ± 1.8) attended two sessions in which they were exposed to a bread odor dispersed, in a detectable but mild concentration, in the test room (“scented” condition) and to a control condition (“unscented” condition). Participants filled out a questionnaire on general appetite before entering the test room and completed a sensory specific appetite questionnaire (including 12 specific products) about 10 min after entering the test room. After approximately 15 min of exposure, the *ad libitum* intake of a low energy dense food product (vegetable soup) was measured. The “scented” condition significantly (*p* < 0.01) increased the amount of soup eaten compared to the “unscented” condition (466.4 ± 33.1 g; 368.9 ± 33.2 g, respectively). Moreover, the odor exposure induced sensory specific appetite for congruent food products in term of taste and energy density, as well as a significant increase in general appetite scores (*p* < 0.001). In conclusion, ambient odor exposure to a food odor affected the intake of a low energy food in obese women and stimulated appetite for congruent products. This could have important implications for influencing energy intake of individuals.

## Introduction

Obesity is becoming a worldwide health problem due to its link with various pathologies, such as diabetes, cardiovascular diseases and cancer (Scherer and Hill, 2016). The growing prevalence of this disease also reflects important changes in society, with an increased consumption of energy dense, processed foods (Blundell et al., 2005).

Mechanisms underlying food choices, and to what extent the sensory experience of eating can influence eating behavior, are still unclear (Sørensen et al., 2003; McCrickerd and Forde, 2016; Zoon et al., 2016). Some evidence suggests that obese subjects are more prone to be affected by sensory cues than lean subjects (Herman and Polivy, 2008). In particular, individuals characterized by higher body mass index (BMI) are associated with a lower responsiveness to internal stimuli (e.g., hunger and satiation signals) (Zoon et al., 2014) and a higher susceptibility to external stimuli (e.g., smell), that contribute to maximizing their energy intake (Schachter, 1968). In this perspective, “cue reactiveness” to external food stimuli could be a potential predisposing factor for overeating (Schachter, 1968; Schachter and Rodin, 1974).

Among food cues that have a function in eating behavior, food odors have a pre-consumption role, aiding in locating food sources, and anticipating the content of foods we are going to eat by inducing (specific) appetite (Zoon et al., 2016). Moreover, odors can affect food choices in a more effective way outside of awareness than in conditions that allow the odor to be consciously identified (Gaillet et al., 2013; Gaillet-Torrent et al., 2014; Smeets and Dijksterhuis, 2014). For example, Gaillet et al. (2013) found that participants who were placed in a waiting room, scented with a non-attentively perceived pear odor, chose significantly more fruit desserts than subjects who were located in an unscented room. Other researchers showed that odors can stimulate appetite for congruent foods, which are similar not only in taste (Ramaekers et al., 2014a,b) but also in energy density (Zoon et al., 2016).

In light of the above, even if it seems reasonable that food odors could play a role in the regulation of food intake, and consequently energy intake, scientific evidence is scarce to support this hypothesis. Indeed, there appears to be a gap between self-report ratings of eating behavior and actual food consumption (Proserpio et al., 2017a). Indeed, some authors showed a decrease in intake upon odor exposure in a subset of subjects (e.g., restrained eaters) (Coelho et al., 2009), while other researchers found an increased intake (Fedoroff et al., 2003; Jansen et al., 2003; Ferriday and Brunstrom, 2008; Proserpio et al., 2017a) or reported no effect of odor exposure on *ad libitum* intake (Ruijschop et al., 2009; Ramaekers et al., 2014b; Zoon et al., 2014). Only few of these mentioned studies have involved overweight individuals to try to understand the impact of odors in this population. Some authors did not find an effect of odor exposure on intake in overweight adults (Ferriday and Brunstrom, 2011; Zoon et al., 2014), while a marginal increase in food intake among overweight children compared to normal-weight was found (Jansen et al., 2003). However, to the best of our knowledge, no studies have been carried out involving morbidly obese subjects.

From this perspective, the first aim of the present study was to investigate in a group of obese women the influence of ambient odor exposure, as occurring in a natural context, on *ad libitum* food intake. Bread odor was used as sensory cue, and a vegetable soup (low energy dense food) was chosen for the *ad libitum* intake since, in Italy, it is common practice to eat bread and soup together as they match each other with regard to the savory taste. The second aim was to evaluate the effect of odor exposure on sensory specific appetite. We hypothesized that food intake and appetite would be affected upon odor exposure and, thus, that implicit cues could be use as strategies to promote the consumption of low energy dense products.

## Materials and Methods

### Participants

Forty obese women (BMI: 35.1 ± 0.8 kg/m^2^; age: 52.1 ± 2.1 years) were recruited at Istituto Auxologico Italiano (Milan, Italy) to participate in a screening session. A control group of normal-weight subjects was not considered since we were interested in deepening the study about the influence of odor exposure toward low energy dense foods, promoting healthy eating in morbidly obese subjects. Thus, a comparison between two BMI groups would not have added information accordingly to the aim of the present study (Bowen et al., 2007; Pol et al., 2018). Moreover, only obese women were recruited in the current research since previous studies suggested the existence of sex-related differences in the perception of odors, reporting that women are usually more sensitive to odors than men and outperforming them in olfactory function (Brand and Millot, 2001; Lundstrom et al., 2005; Hummel et al., 2007; Proserpio et al., 2017b). During the screening session, women were tested with the identification part of the Sniffin’ Sticks task to ensure that they were normosmic (75% correct) (Hummel et al., 2007). Other exclusion criteria were: age >65 years, medical treatment that could modify taste and odor perception, food allergies or intolerances and habitual smoking. Subjects who did not like the odor or the test meal chosen in the study (<40 mm on a 100 mm Visual Analog Scales: VAS, anchored at the extremes “I don’t like it at all”: rated 0, to “I like it a lot”: rated 100) were excluded, so as not to affect negatively the behavioral responses. On the basis of the screening session, ten obese women were excluded mainly due to low liking scores for the test meal or due to less than 75% correct responses in the Sniffing’ Sticks task.

After the screening session, thirty obese women (BMI: 34.9 ± 0.8 kg m^-2^; age: 50.8 ± 1.8) were included in the experimental sessions. In order to keep participants unaware about the real purpose of the experiment, a cover story was told. They were informed that the aim of this study was to improve the sensory quality of a vegetable soup. This study was approved by the Ethic Committee of the IRCCS Istituto Auxologico Italiano, and written informed consent was obtained from all participants after full explanation of the study protocol. The study was performed according to the principles established by the Declaration of Helsinki.

### Measurements

#### Olfactory Stimulus

The participants were exposed to a bread odor (Prolitec Fragrance, Seattle, WA, United States) that was dispersed in the test room using a vaporizer (Prolitec Air/Q 570, WA, United States), which was set to release it in a detectable but mild concentration (one 15-s puff of odor every 2 min), as determined by a pilot study involving 35 subjects. The subjects involved in the pilot study had to indicate the intensity of the ambient odor (100 mm VAS, anchored at the extremes “not at all”: rated 0, to “very”: rated 100) and categorize it into odors signaling a low or high energy dense, and a sweet or savory food product. The results showed that the odor was perceived as detectable but mild (45.4 ± 1.4 on the VAS scale) and was categorized correctly as high-energy dense and savory by 62% of the participants. The pleasantness of the bread odor was also evaluated (63.3 ± 2.7, 100 mm VAS, anchored at the extremes “I don’t like it at all”: rated 0, to “I like it a lot”: rated 100). These subjects were not included in the experimental sessions.

#### General Appetite

To ensure that participants were in a similar hunger state in the two conditions (“scented” and “unscented”), they were asked to rate their appetite at the beginning of each session by filling out a questionnaire on general appetite (hunger, fullness, desire to eat, and thirst) measured on 100 mm VAS, anchored at the extremes “not at all” to “very.”

#### Specific Appetite Ratings

After approximately 10 min of odor exposure, participants rated how much they would want to eat, at that moment, 12 different food products representing four different food categories (all measured on 100 mm VAS, anchored at the extremes “not at all” to “very”). The names of 12 products were given in a randomized order. Participants had to rate how much they would want to eat: 3 high-energy dense sweet products (HDSW: ice cream, cake, and chocolate), 3 high-energy dense savory products (HDSA: breaded veal cutlet, cheese and French fries), 3 low-energy dense sweet products (LDSW: melon, apple, and strawberries), and 3 low-energy dense savory products (LDSA: tomato, zucchini, and raw carrot).

#### Food Intake

During the screening session, the liking for two different vegetable soups (carrot soup and zucchini/potato soup; Zerbinati, Casale Monferrato, Alba, Italy) was measured. The zucchini/potato soup (ingredients: water, zucchini 36%, potatoes 11%, carrots, onions, leeks, olive oil 2%, salt), was preferred in the screening session, and was therefore chosen as food for *ad libitum* intake in the experimental sessions. Food intake (g) was measured after about 15 min of “scented” and “unscented” condition. In both conditions, the participants were given a fixed portion (620 g; 23 kcal/100 g) of vegetable soup to consume. They were instructed to eat the product until they no longer wished to do so and to consume water only at the end. If participants finished the 620 g portion they were provided with another portion. The subjects were unaware that the portion was weighed before and after the test session to determine food intake.

### Experimental Procedure

Each participant was tested on two separate days at the same time (11:30–13:00) with at least 1-day wash-out period between their sessions. In the two test sessions, the participants were asked to refrain from consuming anything but water for 3 h before the test session (hungry state). On 1 day, they were unconsciously exposed to the smell of bread (the “scented” condition) and on the other day, they were not exposed to the odor (the “unscented” condition). The ordering of the “scented” and “unscented” condition was counterbalanced across participants.

Before entering in the test room, participants filled out the questionnaire on general appetite. Subsequently, participants entered in the test room where they were exposed to the “scented” or “unscented” condition. They had to fill in the specific appetite questionnaire 10 min after entering the odorous room. After approximately 15 min of exposure, the participants were entered in a non-odorous room where *ad libitum* food intake was measured, providing the vegetable soup.

### Data Analysis

In order to check if participants were in the same hunger status before the “scented” or “unscented” condition, a paired samples t-test was performed to compare the general appetite ratings (100 mm VAS: hunger, fullness, satiety, desire to eat, and thirst).

To determine the influence of odor exposure on food intake, a linear mixed model was constructed with *ad libitum* intake of soup (g) as dependent factor, and “exposure” (“scented” and “unscented” conditions), as fixed factor. In order to evaluate the influence of odor exposure on sensory specific appetite a linear mixed model was performed by adding specific appetite ratings (100 mm VAS) as dependent factors and “exposure,” “product category” (HDSW, HDSA, LDSW, and LDSA), and their interactions as fixed factors. To check for possible confounding or modulating effects, “session order” (the order of odor conditions: scented 1st and unscented 2nd vs. unscented 1st and scented 2nd) was added as covariate to all the models. Participants were added as random effect. When a significant difference (*p* < 0.05) was found, least significant difference (LSD) *post hoc* test was used. These statistical analyses were performed using IBM SPSS Statistics for Windows, Version 24.0 (IBM Corp., Armonk, NY, United States).

## Results

### General Appetite

The baseline general appetite ratings (Table 1) confirmed that feelings of hunger, fullness, desire to eat, and thirst were not significantly different at the start of the two conditions (“scented” and “unscented”).

**Table 1:** General appetite ratings (means ± SEM), as measured on 100 mm VAS, provided before “scented” and “unscented” conditions by participants.

	Before “scented” condition	Before “unscented” condition	*t*	*p*
Hunger	65.7 ± 3.5	67.9 ± 4.5	0.4	0.7
Fullness	25.9 ± 4.5	28.3 ± 5.0	1.2	0.2
Desire to eat	67.9 ± 5.3	68.6 ± 4.6	0.1	0.6
Thirsty	46.6 ± 3.9	49.5 ± 5.1	0.4	0.9


### Food Intake

**FIGURE 1:**
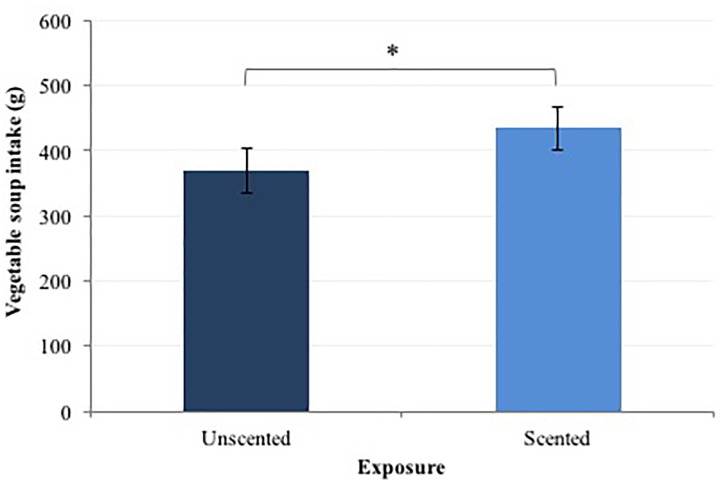
Mean total amount of vegetable soup (in g) eaten *ad libitum* after 15 min of odor exposure (error bars showing SEM). Significant differences in intake between conditions are indicated by ^∗^.

There was a significant effect of “exposure” on participants’ food intake [*F*_(1,29)_ = 8.5; *p* < 0.01]. Figure 1 shows that subjects ate significant larger amount of soup during the “scented” than during the “unscented” condition (466.4 ± 33.1 g vs. 368.9 ± 33.2 g), and this was not influenced by “session order.”

### Sensory Specific Appetite

Table 2 shows that there was a significant effect of “exposure” on specific appetite scores [*F*_(1,674)_ = 22.4, *p* < 0.001], with generally higher scores provided during the “scented” (45.7 ± 2.5) compared to the “unscented” condition (35.5 ± 2.6).

**Table 2:** Specific appetite scores (means ± SEM), as measured on 100 mm VAS, for the product categories (HDSA, LDSA, HDSW, and LDSW) during the “scented” and “unscented” condition.

Sensory specific appetite	“Scented” condition	“Unscented” condition
HDSA	52.7^a^ ± 3.7	31.6^a^ ± 3.7
LDSA	46.3^ab^ ± 3.6	34.7^a^ ± 3.8
HDSW	44.3^ab^ ± 3.7	36.4^a^ ± 3.6
LDSW	39.6^b^ ± 3.7	39.5^a^ ± 3.5


*Different superscript letters within columns indicate significant differences (*p* < 0.05).*

Moreover, there was a significant interaction between “exposure” and “product category” [*F*_(3,674)_ = 4.1; *p* < 0.01] indicating sensory specific appetite. *Post hoc* comparison revealed that during the “scented condition” appetite for HDSA products significantly increased compared to the other product categories. Similar to *ad libitum* intake, these results were not affected by “session order.”

## Discussion

The present study showed that ambient odor exposure increased the consumption of vegetable soup in morbidly obese women and induced sensory specific appetite.

These findings confirm previous observations in normal-weight women in a similar setting (Proserpio et al., 2017a). In particular, in the mentioned study the exposure to odors signaling high-energy dense products (e.g., chocolate and beef), increased food intake, and also salivation, compared to control condition (no odor exposure). Contrary to our findings, Ferriday and Brunstrom (2011) showed an effect of the exposure to the sight and smell of pizza on overweight participants’ desire to eat, but not on actual food intake, showing a gap between explicit, self-report ratings (appetite scores) and more implicit measurements (food intake). These differences between studies could be due to the different concentrations of the odors and thus differences in awareness of the subjects toward the food cues. Indeed, it has been reported that food choices are driven mainly by non-conscious processes (Gaillet-Torrent et al., 2014) and eating behavior is likely influenced to a larger extent by odors presented outside of subject’s awareness than in conditions in which it is clearly possible to recognize them (Smeets and Dijksterhuis, 2014). According to this hypothesis, Zoon et al. (2014) did not found an effect of odor exposure at clearly noticeable intensities on food consumption in overweight women.

Beside the effect of odor exposure on *ad libitum* food consumption, the current study showed that bread odor increased not only generally appetite, but also sensory-specific appetite, for food products that are similar both in taste and in energy density. In particular, appetite for HDSA increased significantly after smelling an odor that signals savory products. These findings, in line with previous studies showing that odors can specifically induce appetite for the cued foods and similar products (Ramaekers et al., 2014a,b; Zoon et al., 2016), highlight that this effect is not restricted to healthy-weight participants, but also extends to morbidly obese women. The increase in appetite for congruent foods in taste and energy density after specific food odor exposure is consistent with the theory that sensory (odor) food cues predict the macronutrient content and prepare the body for intake (Brunstrom, 2007; Brunstrom and Mitchell, 2007). These associations are most often described in terms of taste quality: sweet taste is related to high-carbohydrate content, while savory taste is associated with high-protein content (Luscombe-Marsh et al., 2008; Viskaal-van Dongen et al., 2011; Berthoud et al., 2012).

Food intake is largely determined by hunger and satiation (Sørensen et al., 2003; Berthoud, 2004), however, in obese individuals, external signals of reward such as olfactory cues of food can override the internal signals of hunger and satiety (Herman and Polivy, 2008; Small, 2009). In this context, the “externality-theory” originally proposed by in Schachter (1968), postulates that obese subjects are more susceptible to food external stimuli, influencing their attitude toward foods and leading them to increase their craving for foods, being prone to overeat.

Appetizing olfactory food cues are part of our current environment that promotes overconsumption, ultimately contributing to a higher incidence of nutrition-related diseases (e.g., obesity and diabetes). Present results suggest that exposure to a food odor impacts actual eating behavior of obese women, with the promising possibility of increasing intake of healthy and low-energy dense foods, which would be suitable as part of a balanced diet.

Some limitations should be mentioned. We examined the effects of the scented condition only on actual food intake of a given product, and not on food choices that typically precede consumption in real life. Future research is necessary to study the impact of odor exposure on food choices, for instance by giving subjects the possibility to choose between different dishes that differ in level of congruency (taste and/or energy-density) with the odor. Moreover, we only included obese women, limiting the ability to generalize our findings to obese men.

Future studies are needed to investigate whether the strategy of odor exposure may be used to reduce the intake of high-energy dense products in obese subjects. Moreover, as odor exposure appears to increase the appetite for congruent products, similarly food odors could be used to stimulate appetite of individuals who suffer from a lack of appetite or are undernourished (e.g., elderly people and anorexic subjects).

In conclusion, the findings of this study may have important implications for improving the compliance of obese subjects to a low caloric diet. Indeed, it could be hypothesized that odor exposure could be used to direct obese subjects toward the consumption of low energy healthy foods, which are generally less appreciated than high energy dense foods, and maybe steer subjects’ food intake away from less healthy choices.

## Author Contributions

CP and EP designed the study. CP and LP carried out the experiment. CP performed the statistical analysis. CP and CC wrote the original draft of the manuscript. CP, CI, SB, ML, CC, and EP regularly discussed the experiments, analyzed the results, and provided the useful suggestion during the writing. ML, CI, SB, and EP reviewed the manuscript. All authors read and approved the final manuscript.

## Conflict of Interest Statement

The authors declare that the research was conducted in the absence of any commercial or financial relationships that could be construed as a potential conflict of interest.
